# Sunitinib and TB lymphadenitis: An unexpected link in oncological therapy: a case report

**DOI:** 10.1097/MD.0000000000043158

**Published:** 2025-07-18

**Authors:** Mi-Ran Han, Kyu-Yun Jang, So-Yeon Jeon

**Affiliations:** a Division of Hematology/Oncology, Department of Internal Medicine, Jeonbuk National University Medical School, Jeonju, Republic of Korea; b Research Institute of Clinical Medicine of Jeonbuk National University – Biomedical Research Institute of Jeonbuk National University Hospital, Jeonju, Republic of Korea; c Department of Pathology, Jeonbuk National University Medical School, Jeonju, Republic of Korea.

**Keywords:** sunitinib, TB lymphadenitis, tyrosine kinase inhibitor

## Abstract

**Rationale:**

Gastrointestinal stromal tumors (GIST) are neoplasms that originate from the interstitial cells of cajal located in the muscular layer of the gastrointestinal tract. Surgery is the primary treatment options; however, if resection is not feasible, or in cases of metastatic or recurrent GIST, systemic chemotherapy can be considered as an alternative. Sunitinib, a multi-targeted tyrosine kinase inhibitor, is indicated as an essential second-line treatment for GIST following disease progression or intolerance to imatinib mesylate. It works by slowing down or stopping the growth of tumor cells through the inhibiting tyrosine kinases, including KIT and PDGFRα. Commonly reported side effects of sunitinib include hypertension, fatigue, neutropenia, and dermatologic reactions such as rash.

**Patient concerns:**

A patient diagnosed with relapsed malignant gastrointestinal stromal tumors (GIST) was treated with sunitinib for 15 months following the failure of imatinib treatment. The patient presented with neck pain and a fever of up to 38.8°C.

**Diagnoses and interventions:**

Upon evaluation, a palpable lymph node was biopsied, and pathology results confirmed tuberculosis (TB) lymphadenitis. Subsequently, TB medication was initiated, and 2 months after starting the treatment, significant improvement in lymphadenopathy was observed on the computed tomography scan. However, the patient experienced side effects during the treatment, including hepatotoxicity, visual disturbances, and a decreased platelet count, which led to discontinuation and a change in medication.

**Outcomes:**

The treatment lasted for a year, which was longer than that initially planned. Despite switching to third-line therapy for GIST, the disease progressed, and the patient eventually died.

**Lessons:**

Owing to the anti-angiogenic effect of sunitinb, infectious complications are very rare, and cases of tuberculosis-related side effects associated with sunitinib are almost unheard of. This case illustrates that TB lymphadenitis can occur as a rare adverse effect of sunitinib treatment.

## 1. Introduction

Gastrointestinal stromal tumors (GIST) are mesenchymal tumors derived from Cajal cells, primarily located in the small intestine and stomach.^[1]^ The treatments for GIST include surgical resection, targeted therapy, and radiation therapy. Molecular targeted therapy is particularly recognized as the cornerstone of GIST treatment, with proven therapeutic effects. If surgery is not feasible, or in cases of metastatic or recurrent disease, or when there is a high risk of recurrence, or in the presence of genetic mutations, tyrosine kinase inhibitor (TKI) chemotherapy should be considered.

Among them, sunitinib is known as an effective drug for GIST, functioning as a multi-TKI that suppresses angiogenesis and growth within tumors.^[2,3]^ In GIST, sunitinib is used as a second-line chemotherapy when it is refractory to or relapses after imatinib therapy. The side effects of sunitinib are commonly known as gastrointestinal disturbances such as nausea and diarrhea, hypertension, skin rash, neutropenia and lymphopenia.^[4]^ As it primarily acts through antiangiogenesis, despite causing neutropenia, its direct association with infections is not well-established.^[5]^ We report here an uncommon case of TB lymphadenitis occurring after the use of sunitinib in a relapsed GIST patient.

## 2. Case presentation

An 80-year-old woman undergoing treatment for GIST with peritoneal seeding presented to the emergency room with a fever of up to 38.8°C. The patient was initially diagnosed 3 years ago and was treated with imatinib for 2 years. However, imatinib was discontinued due to signs of disease progression, and the patient had been taking sunitinib since then. Upon admission, she complained of severe neck pain. During the physical examination, erythematous color changes and tender masses were observed around the cervical area, with a fever measured above 38°C. Six months ago, the patient had visited the emergency room due to pain and enlarged lymph nodes on the right side of the neck, similar to the current symptoms. She had visited the otolaryngology department, where a 1.5 cm hard and mildly tender lymph node had been noted. At that time, computed tomography (CT) and aspiration cytology had revealed the diagnosis of cervical lymph node metastasis at the right neck level. However, in this case, there was accompanying fever, and the intensity of tenderness was more severe than before. The CT scan also showed a necrotic mass with unclear borders around the right sternocleidomastoid (SCM) muscle. Due to different clinical symptoms and imaging results from before, a tissue biopsy was repeated for further evaluation.

**Figure 1.**
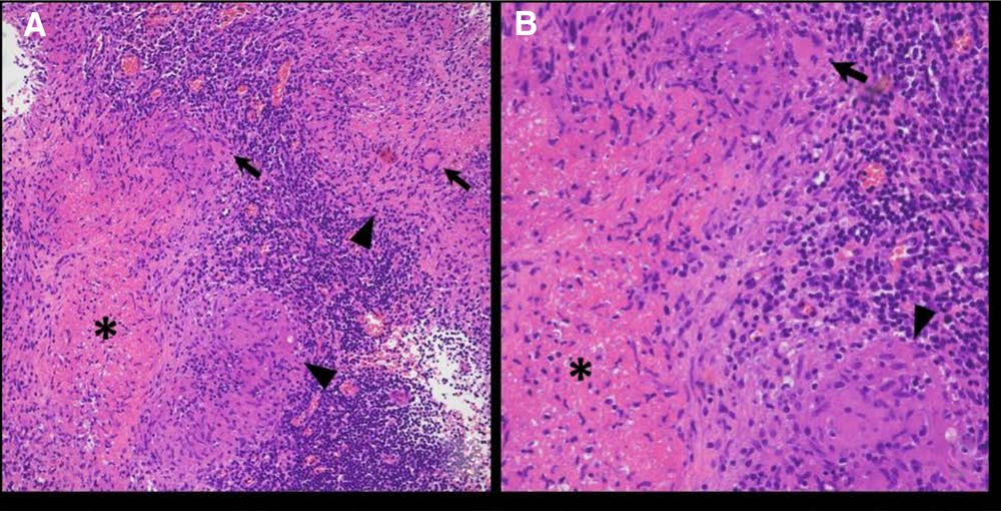
Histologic findings of Mycobacterium tuberculosis. (A and B) Chronic granulomatous inflammation composed of granulomas (arrowheads), Langerhans giant cells (arrows), and caseation necrosis (asterisks). Original magnification: (A) ×200 and (B) ×400.

**Figure 2.**
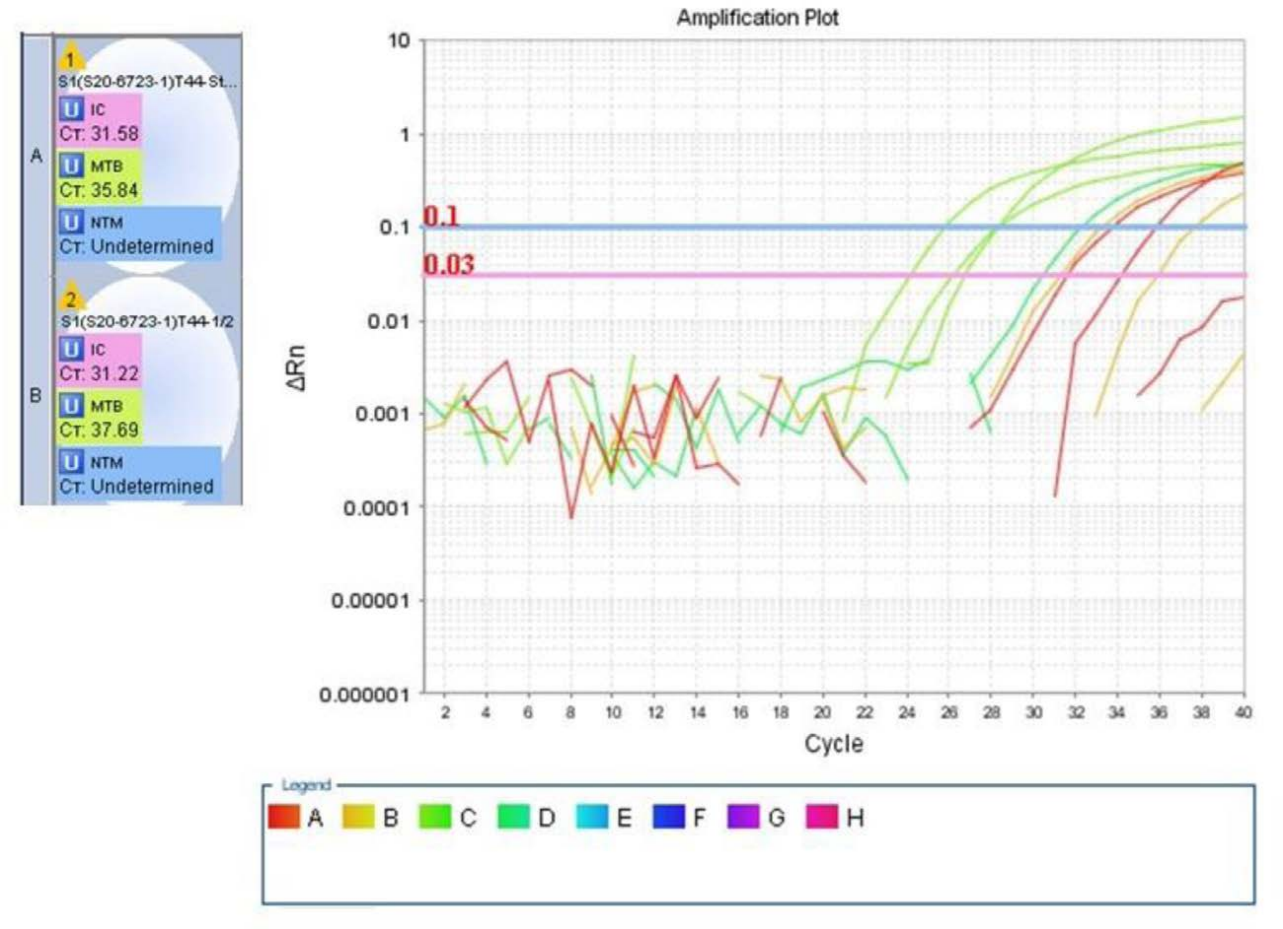
A positive TB PCR positive result. The Ct values that cross the threshold line for both (A and B) are within the reference range, indicating a positive result. Ct = cycle threshold, PCR = polymerase chain reaction, TB = tuberculosis.

Surprisingly, the final histopathological examination confirmed chronic granulomatous inflammation due to Mycobacterium tuberculosis complex infection (Fig. 1), and tuberculosis (TB) polymerase chain reaction was also positive (Fig. 2). Retrospectively, following the tissue biopsy diagnosis, the patient also had a positive result on interferon-gamma release assay testing. There are no pre-chemotherapy test results available to confirm prior latent tuberculosis based on out tests. However, according to the patient’s history, including family history, there was no prior history of latent tuberculosis. The tissue appearance clearly indicated TB, but immunohistochemical staining for C-kit and discovered on GIST 1, conducted to definitively differentiate it from the previous metastatic lesion, showed a negative result.

**Figure 3.**
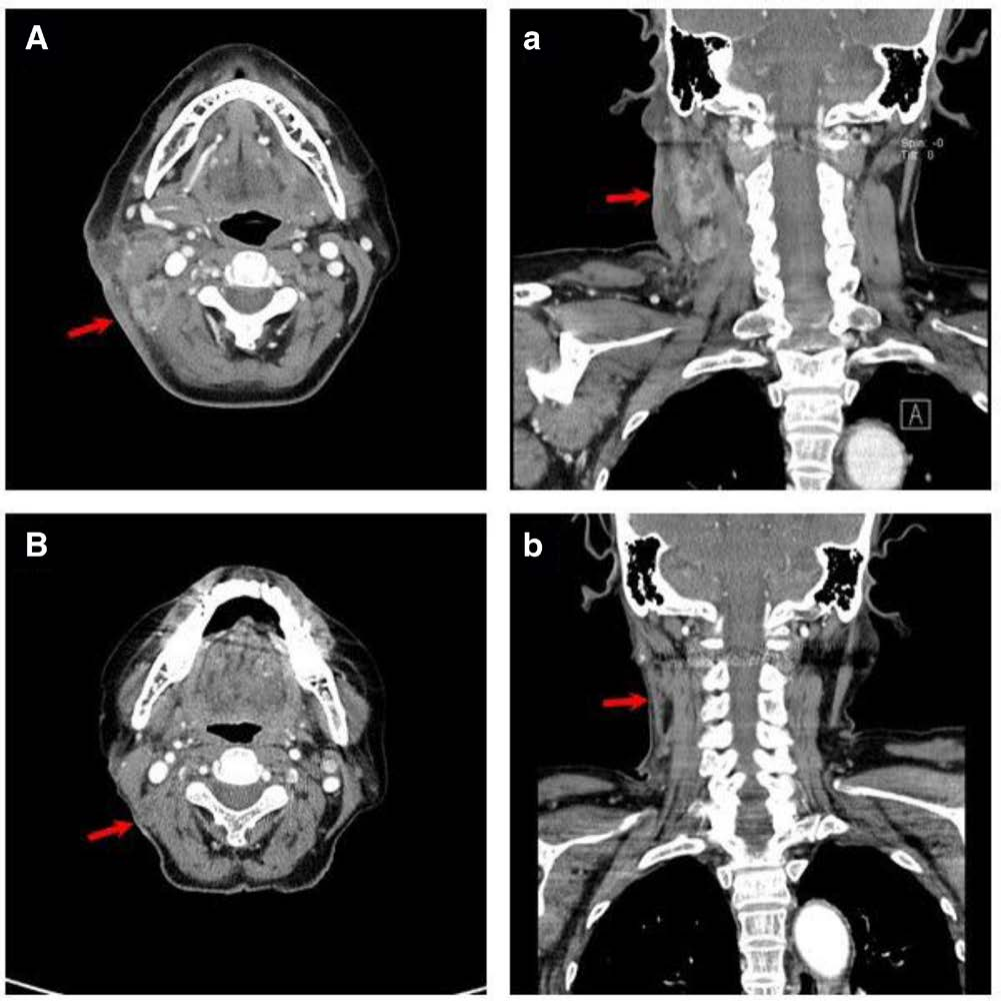
Computed tomography images of neck comparing the cervical lymph nodes before and 2 mo after starting TB medication. (A) A mass with a necrotic center around the right SCM muscle. (B) Posttreatment CT neck image, showing that the necrotic lymph nodes have reduced in size and significantly improved. CT = computed tomography, SCM = sternocleidomastoid, TB = tuberculosis.

We initiated Isoniazid, rifampicin, ethambutol, and pyrazinamide for TB lymphadenitis. One month after starting tuberculosis medication, the TB medication was switched from Isoniazid to levofloxacin due to increased liver function test and suspicion of hepatotoxicity. Subsequently, after experiencing symptoms of thrombocytopenia and blurred vision, the medication was discontinued and changed several times over the course of approximately 1 year. Despite frequent changes in medication due to side effects of the treatment, 2 months after the initiation of TB management, imaging studies and physical examinations revealed significant improvement in lymphadenopathy (Fig. 3).

For malignant GIST, resection was performed due to small bowel bleeding. Four months after starting tuberculosis medication, sunitinib was resumed. However, during the restaging conducted 5 months after resuming chemotherapy, a CT scan revealed an increase in size and enhancement of peritoneal metastasis, indicating progressive disease. Consequently, third-line therapy with regorafenib, another multi-TKI, was initiated. Unfortunately, the disease progressed again after 1 year, prompting a switch to sorafenib. Despite these efforts, the patient’s condition did not improve, and eventually, she passed away.

## 3. Discussion and conclusions

Gastrointestinal stromal tumor (GIST) is a rare tumor with an incidence of 1.2 per 10^5^ individuals per year, originating from the interstitial cells of Cajal in the gastrointestinal tract, found in the mucosa or muscular layers of the stomach, esophagus, or intestines.^[1,6]^ About 80% of GIST is characterized by activating mutations in the tyrosine kinase domains of the KIT or PDGFRα.^[1]^ The size of the tumor and its mitotic count are crucial factors influencing the treatment decision for GIST. Lesions smaller than 2 cm can typically be observed, but those larger than that require surgical resection as a primary treatment. It is known that metastatic relapse occurs in 40% of patients with localized GIST,^[2]^ so adjuvant chemotherapy may be necessary. Additionally, chemotherapy is required for locally advanced patients, neoadjuvant therapy, and unresectable tumors with metastasis. Imatinib is a type of multi-TKIs that blocks the phosphorylation of tyrosine, an amino acid essential for cell signaling, used as first-line in the treatment of GIST. It works by selectively blocking the activity of certain abnormal proteins, such as KIT and PDGFRα, which are often mutated and overactive in GIST.^[7]^ When the disease progresses despite treatment with imatinib or if a patient cannot tolerate it, sunitinib can be used as a second-line chemotherapy agent in the treatment of GIST.

Sunitinib is an oral targeted anticancer agent used in renal cell carcinoma, GIST, non-small cell lung cancer, and other cancers. As 1 of the multi-targeted TKIs, it inhibits specific proteins crucial for the development and progression of tumors such as vascular endothelial growth factor receptors (VEGFR type 1 and 2), platelet-derived growth factor receptors (PDGFRα and β), KIT.^[8]^ Its main action is known to be through its anti-angiogenic effects. The common side effects of sunitinib include hypertension due to its action on VEGFR, along with diarrhea, fatigue, and skin rash.^[9]^ Sunitinib not only affects cancer proliferation and survival but also has effects on the immune system, potentially influencing susceptibility to infections, including bacterial, viral, and fungal pathogens, due to causing severe neutropenia and lymphopenia, through the inhibition of specific lymphocyte subsets.^[10]^ In fact, since sunitinib mainly exhibits anti-angiogenic effects, it can cause cytopenia as a side effect itself. However, its direct association with infections is largely unknown, and cases are rare.^[5]^ Furthermore, cases like this patient’s, where TB has occurred, are even more challenging to identify for academic research. T lymphocytes play a crucial role in defending against Mycobacterium tuberculosis (MTB). Studies have indicated that patients with COVID-19 infection accompanied by lymphopenia are also at an increased risk of latent TB reactivation.^[11]^ Th1 and Th2 cells, subtypes of CD4 + T helper cells, have distinct functions. Th2 cells secrete IL-4 and IL-10 to activate humoral immunity, while Th1 cells secrete IFN-γ and IL-2 to activate macrophages to eliminate MTB. Additionally, CD8 + T cells differentiate into cytotoxic T lymphocytes (CTLs), which ultimately kill infected cells. Lymphopenia may make individuals more susceptible to TB infection. Conversely, TB infection can worsen lymphopenia by inhibiting lymphocyte proliferation, inducing apoptosis and exhaustion of T lymphocytes, and causing bone marrow hematopoietic dysfunction.^[12]^ Upon admission to the emergency room, the patient had a lymphocyte percentage of 9.9% and an absolute count of 710 cells/µL, indicating lymphopenia, defined as a lymphocyte percentage of <20% of the total leukocyte count or an absolute lymphocyte count of <1000 cells/mL. Patients with observed lymphopenia are not only susceptible to infections such as tuberculosis, human immunodeficiency virus, cytomegalovirus, and influenza, but it also suggests that these patients may already be suffering from such infections. Therefore, the association between cytopenia caused by TKI administration and the development of TB cannot be completely overlooked.

In this case, the focus is on tuberculosis (TB) that developed after approximately 1.5 years of sunitinib treatment. Currently, it is not directly known to modulate the immune system, and its association with TB has been rarely reported. While the direct link between multi-targeted TKIs and TB is not well-established, in regions with high TB prevalence like Korea, and particularly in patients with observed lymphopenia, monitoring for latent TB through tests such as IGRA and signs of TB reactivation is warranted when undergoing TKI therapy.

## Author contributions

**Conceptualization:** So-Yeon Jeon.

**Data curation:** Kyu-Yun Jang.

**Supervision:** So-Yeon Jeon.

**Validation:** Kyu-Yun Jang.

**Writing – original draft:** Mi-Ran Han.

**Writing – review & editing:** Mi-Ran Han.
